# Supplementary material for: Conditional chemoconnectomics (cCCTomics) as a strategy for efficient and conditional targeting of chemical transmission
Source: eLife. 2024 Apr 30;12:RP91927. doi: 10.7554/eLife.91927 (PMC11060718; doi:10.7554/eLife.91927)
Supplement: Figure 3—source data 3. [file elife-91927-fig3-data3.zip › Figure 3 source data3 part 1.zip!/Figure 3-Source data 3-part1/1071f3-1-CRISPResso_Report_jvi77nmx.zip!/Nucleotide_percentage_quilt.pdf]

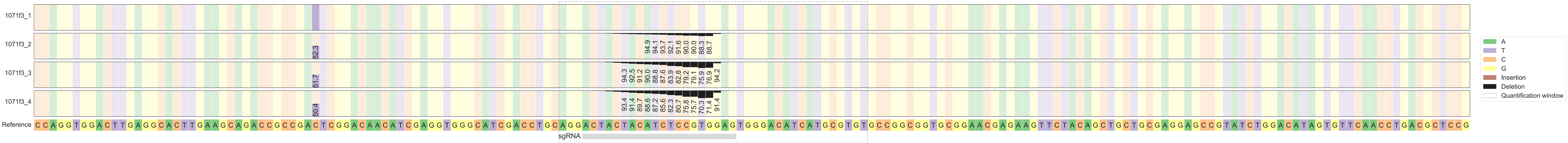

1071f3_1
1071f3_2
52.3
94.9 94.1 93.7 92.1 91.6 90.0 90.0 88.3 88.7
1071f3_3
51.7
94.3 92.5 91.2 90.0 88.8 87.6 83.9 82.8 79.2 79.1 75.9 76.9 94.2
1071f3_4
50.4
93.4 91.4 89.7 88.6 87.2 85.6 82.3 80.7 75.8 75.7 70.3 71.4 91.4
Reference
CCAGGTGGACTTGAGGCACTTGAAGCAGACCGCCGACTCGGACAACATCGAGGTGGGCATCGACCTGCAGGACTACTACATCTCCGTGGAGTGGGACATCATGCGTGTGCCGGCGGTGCGGAACGAGAAGTTCTACAGCTGCTGCGAGGAGCCGTATCTGGACATAGTGTTCAACCTGACGCTCCG
sgRNA
A
T
C
G
Insertion
Deletion
Quantification window
